# A review of assistive product prices in 12 countries

**DOI:** 10.3389/fresc.2026.1736102

**Published:** 2026-04-29

**Authors:** Natália Hedlund Jardim, Vinicius Delgado Ramos, Irene Calvo, Johan Borg, Kylie Shae

**Affiliations:** 1 Instituto de Medicina Física e Reabilitação, Hospital das Clinicas HCFMUSP, Faculdade de Medicina, Universidade de Sao Paulo, Sao Paulo, Brazil; 2 Medical Devices and Assistive Technology Team, Health Systems, Access and Data Division, World Health Organization, Geneva, Switzerland; 3 School of Health and Welfare, Dalarna University, Falun, Sweden

**Keywords:** affordability, assistive product, assistive technology, cost, price, priority assistive products list

## Abstract

**Purpose:**

The World Health Organization (WHO) and the United Nations Children's Fund (UNICEF) estimate that over 2.5 billion people need assistive technology, yet access remains limited. In response to this pressing need, WHO has maintained, since 2016, a Priority Assistive Products List (APL) with 50 priority assistive products. In 2024, an update was launched to revise and expand the list based on new evidence and stakeholder input. This paper presents the price review component of the update. The review consisted of collecting global price data and classifying assistive products into price ranges to support the decision-making process for the updated APL.

**Materials and methods:**

From an initial list of 300 products prioritized by the WHO Technical Advisory Group on Assistive Technology, 120 products were selected for the price collection by domain experts. Twelve countries, representing a range of income levels and geographic regions, were chosen for data collection. Focal points in each country gathered the lowest prices for the selected assistive products. The collected price data was then used to classify the products into five Gross Domestic Product-standardized price ranges.

**Results:**

Focal points from all 12 countries submitted price data to varying extents. In eight countries, the data covered over 75% of products, whereas in the remaining four countries, the coverage was below 50%, as focal points faced challenges such as limited supplier access or time constraints. The collected data provided insights into the affordability of assistive products across countries and product categories.

**Conclusion:**

The price analysis contributed essential evidence to the APL update and highlighted global disparities in affordability of assistive products. The data showed an inverse relationship between national income level and affordability, where the lower the income, the greater the financial burden assistive products represent.

## Introduction

The World Health Organization (WHO) and the United Nations Children's Fund (UNICEF) estimate that over 2.5 billion people worldwide require assistive technology, yet access remains critically low in some regions, with coverage as limited as 3%. Access to assistive products is a fundamental human right, essential for enabling individuals to live productive, dignified, and independent lives (1).

In response to this pressing need, WHO has maintained, since 2016, a Priority Assistive Products List (APL) comprising 50 priority assistive products that support functioning in cognition, communication, hearing, mobility, self care and continence, and vision. The APL is intended to serve as a model for WHO Member States in developing their own national lists of priority assistive products, tailored to local needs and available resources. Additionally, it aims to stimulate product development and manufacturing, guide service delivery and procurement strategies, inform market-shaping and reimbursement policies, and support broader health system planning (2).

The APL is part of a wider effort to improve access to assistive technology globally. In its first-ever resolution on the subject, the World Health Assembly called upon Member States to “develop a national list of priority assistive products that are affordable and cost-effective and meet minimum quality and safety standards, drawing on WHO's Priority Assistive Products List” (3). More recently, the WHO–UNICEF *Global report on assistive technology* reiterated this call, highlighting the significant role the WHO APL can play in measuring access to assistive products, building the capacity of human resources to provide assistive technology, and facilitating the collaborative efforts of various stakeholders to ensure that assistive products are accessible to everyone in need (4).

In light of the *Global report* published in 2022 and recent technological advancements, WHO launched an effort to update the 2016 APL. This revision aimed to incorporate new evidence to review and expand the list with additional prioritized assistive products through a transparent and systematic process. The update involved evidence reviews, price analyses, and stakeholder consultations to support informed decisions on the inclusion or exclusion of specific assistive product types. To support this effort, the WHO Access to Assistive Technology (ATA) team coordinated with experts and organizations in the field to collect and analyze data that would inform the review process.

In collaboration with methodologists, the WHO Technical Advisory Group on assistive technology, the APL Steering Committee, and other stakeholders, WHO defined four key indicators to guide the prioritization of products in the APL: *need*, *benefits*, *risks*, and *price*. This paper focuses on the review of the final indicator—*price*. The review informed the APL update by classifying each assistive product into a price range, providing the WHO ATA team with a clearer understanding of global price levels for assistive products.

## Materials and methods

To conduct the review, WHO engaged the *Physical and Rehabilitation Medicine Institute* (IMREA) of the *University of São Paulo Medical School General Hospital*. Under the supervision of the WHO ATA team, IMREA coordinated a seven-month project, from October 2024 to April 2025, to collect and analyze assistive products price data across 12 countries. The review was conducted by a multidisciplinary team that included six domain experts and 17 volunteer focal points responsible for collecting national-level price data. Relevant steps of the methodology adopted for the price review are described in greater detail in the subsections that follow.

### Selection of priority products and specifications

The WHO ATA team, with support from the Technical Advisory Group on assistive technology conducted a screening of the ISO 9999:2022 standard on *Assistive products for persons with disability—Classification and terminology*, which establishes a classification and terminology of assistive products (4). During this screening, the original 948 divisions defined in the standard were reduced to 300. This reduction focused on identifying divisions most relevant to the project and excluding those that did not align with the WHO definition of assistive products (2) – such as medical devices, therapeutic products, or accessories.

Due to resource constraints, WHO and IMREA considered it unfeasible to collect price information for all 300 products. Therefore, the IMREA team carried out a preselection process guided by two criteria: maintaining the original 50 products included in the 2016 APL and including at least one product from each of the 64 ISO 9999:2022 subclasses (the second level of the classification system, which provides more specific groupings under the main classes), ensuring representation across the six functional domains.

Grouping assistive products pertaining to diverse ISO 9999 classes around the six domains of the APL often demanded discretionary choices aimed at guaranteeing greater diversity of products included. Many ISO classes and subclasses could easily fit more than one domain, such as with assistive products for communication and information management (class 22) and for recreation and leisure (class 30). Whenever possible, the reference product selected for such classes provided a bridge between these two realms. This was the case with selecting a calendar clock for people with Alzheimer's or dementia for Calendars and timetables (22 28 06) or tactile dominoes for Games (30 03 09), as per the steps described below.

**Figure 1:**
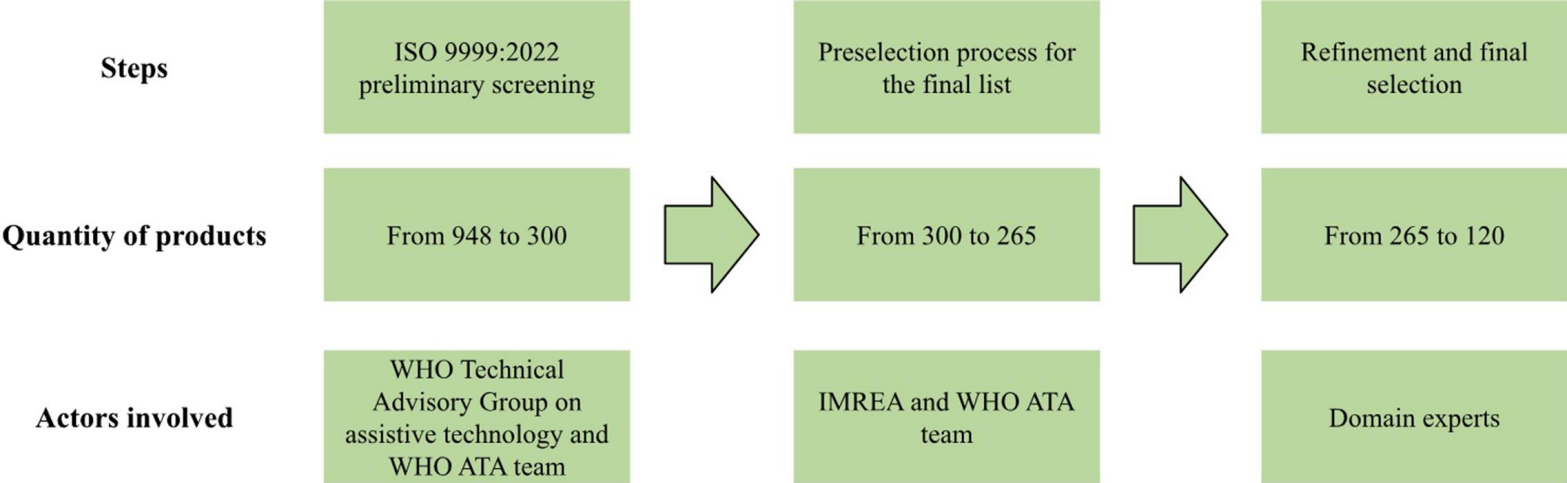
Overview of the selection process for priority products.

This pre-selected list was then shared with six domain experts in assistive technology, who refined it and selected 120 proposed products for which price data would be collected. The domain experts were experienced practitioners with expertise in the provision and servicing of assistive products and were selected to provide technical guidance throughout the project. Each expert specialized in one of the six functional domains: cognition, communication, hearing, mobility, self care and continence, and vision. Their selection prioritized relevance to each functional domain and the potential to benefit a broader population of assistive product users. Figure 1 below illustrates the selection process. The final list is available in Supplementary Table.

Once the list of 120 products was approved by the WHO ATA team, the domain experts proceeded to identify one reference product for each listed item. These reference products were, whenever possible, off-the-shelf, regulatory-compliant products available through global suppliers. It is important to note that referencing specific companies or manufacturers did not imply endorsement or preference over similar, unlisted alternatives. Rather, these example products aimed to support comparability across settings—particularly for identifying products with similar service delivery needs.

For each reference product, domain experts provided a web link to technical specifications and indicated whether accessories or consumables were needed. When applicable, they also identified reference products for those additional items and included their respective web links. This information—including reference products, web links, accessory and consumables details—was submitted through an online form completed by each domain expert. The IMREA team compiled the submissions and presented the finalized dataset to the WHO ATA team for validation.

### Country selection

Concurrently with the selection of the priority products, the IMREA team, with support from the WHO ATA team, selected 12 target countries for the price collection exercise, ensuring diversity in both geographical distribution and income level. The selection was guided by two main criteria:

1. **Income level distribution**, ensuring representation in line with the proportion of countries within each World Bank income classification (Low, Lower-middle, Upper-middle, and High).
2. **Geographic distribution**, based on the six WHO global regions: African Region, Eastern Mediterranean Region, European Region, Region of the Americas, South-East Asia Region, and Western Pacific Region.

The final list of selected countries is presented in Table 1, below.

**Table 1:** Selected countries for the price review.

Country	World Bank income level	WHO region
Brazil	Upper-middle income	Region of the Americas
Canada	High income	Region of the Americas
China	Upper-middle income	Western Pacific Region
India	Lower-middle income	South-East Asia Region
Italy	High income	European Region
Japan	High income	Western Pacific Region
Mozambique	Low income	African Region
Norway	High income	European Region
Senegal	Lower-middle income	African Region
Sudan	Low income	Eastern Mediterranean Region
Thailand	Upper-middle income	South-East Asia Region
Zambia	Lower-middle income	African Region

### Price Review Committee

The Price Review Committee comprised 17 volunteer focal points who participated in their personal capacity to collect price data in the selected countries. Each focal point had extensive experience in and familiarity with one of the 12 target countries, especially with the local provision of assistive products. To establish the committee, WHO and IMREA shared targeted invitations through their networks to encourage volunteers to take part in the process.

Committee members were invited to participate based on a set of non-exclusive criteria, including: relevant technical expertise, experience in international and country-level policy work, strong communication skills, and the ability to collaborate effectively with individuals from diverse cultural backgrounds. Participation was voluntary, and members were not remunerated for their involvement. The focal points were also asked to disclose any potential conflicts of interest.

The core responsibility of the Price Review Committee was to identify comparable, regularly available assistive products for each of the 120 priority products and to collect corresponding price data—including for any applicable accessories and consumables—within the 12 selected countries. For each entry, members were required to indicate the source of the price and, whenever possible, provide a reference web link. Each member collected price data for one target country. Japan had two focal points conducting the price collection, Mozambique and Senegal had three each, and the remaining countries had one.

Recruitment for the committee began in late November 2024 and continued through early March 2025. Two initial training sessions were held on January 9 and 10, 2025, during which the Price Review Protocol and project methodology were presented to focal points from Brazil, Canada, China, India, Italy, Japan, Norway, Senegal, and Thailand. Separate training sessions were later conducted for focal points from Mozambique, Zambia, and Sudan.

During the price collection phase, two focal points withdrew from the project due to their unavailability to continue supporting the price collection activities. This led to the recruitment of a new focal point for India and the replacement of Ghana with Zambia in the final list of selected countries. After the training sessions, focal points had three months to conduct the price collection, with the exception of Mozambique, which had two months, and India, Sudan, and Zambia, which had one month due to their later recruitment.

### Price collection

The price collection process began following the training sessions conducted with the focal points. Each focal point received an online price review form listing all 120 priority assistive products, along with their respective reference products, specification links, accessories, and consumables (when applicable).

The focal points were asked to identify products that were regularly available and regulatory compliant within their respective countries—closely matching the reference products. For the purpose of the price collection, “regularly available products” were defined as products locally accessible through providers of assistive technology, suppliers of assistive products, or the open market; products requiring special importation or purchase from another country for the specific purpose of data collection were not considered in the price collection.

To gain an indication of product prices across countries, the lowest available price of a regulatory-compliant product in each country was used. This approach was selected because comprehensive price collection, covering all models and variants, would not be feasible and would produce averages that do not meaningfully represent what is actually provided. Additionally, the purpose of price data collection was not to inform budgeting, but rather to compare product categories relative to each other. In this instance, products with the lowest price in each country were likely to be more comparable across settings than higher-priced alternatives.

Therefore, for each product, the focal points were instructed to collect the lowest available price in United States dollars (USD), using official exchange rates (e.g., those provided by national banks) for currency conversion at the time of data collection. They were also asked to indicate the source of the price, selecting from the following categories: provider, supplier, open market, custom-made, not available, or other. The same procedure applied to accessories and consumables, when applicable. For consumables, the annual price was requested. At the end of the form, the focal points were also asked to provide a reference web link to the product from which the price was obtained.

The priority during price collection was to collect the lowest price (including applicable taxes) without services that a government or government approved provider or procurement agency needs to pay a supplier. If the product is not provided by a provider, or not covered by a purchase agreement with a provider, the second option was to collect the lowest price (including applicable taxes) directly from suppliers. If that too was unavailable, the third option was to obtain the lowest price from the open market. If a regulatory compliant assistive product, or any of its accessories or consumables, was not regularly available in a given country, no price or service life information was to be reported for that product.

During the price collection phase, the domain experts worked concurrently to review the data submitted by the focal points. They ensured that the listed products met the minimum technical specifications of the reference models and that the reported prices were realistic and aligned with market standards. When necessary, feedback was shared with the focal points, who revised the data based on the experts' observations.

### Price calculations

Based on the collected prices, the annual price was calculated for each product across all reporting countries, using the following formula:

$$\begin{array}{rl} \mathrm{Price}\;\mathrm{Annual}= & \left(\frac{\mathrm{Price}\;\mathrm{Product}}{\mathrm{Service}\;\mathrm{Life}\;\mathrm{Product}}\right)+\left(\frac{\mathrm{Price}\;\mathrm{Accessories}}{\mathrm{Service}\;\mathrm{Life}\;\mathrm{Accessories}}\right)\\ & +\mathrm{Price}\;\mathrm{Annual}\;\mathrm{Consumables}. \end{array}$$

Only products available in the countries and for which prices were collected were included in the calculation. Some products were reported as having no cost—for example, online apps or software for digital accessibility that can be downloaded or used for free. When focal points did not report a product's service life, the median service life from the other countries that did report it was used. Additionally, products of a disposable nature that require multiple units per year—specifically those in ISO divisions 09 06 06, 09 24 03, 09 24 06, 09 24 09, 09 30 13, 09 30 18, 09 30 21, 09 30 26, and 09 30 45 (see Annex for details)—had their service life estimated based on annual projections provided by the domain experts.

Using the annual prices, the median annual price for each product was determined across all reporting countries. Additionally, to compare the prices among different product categories, the median standardized price was calculated based on each country's reported 2023 Gross Domestic Product (GDP) per capita (as published by the World Bank). To calculate the GDP-standardized price, the following formula was used:

$$\mathrm{GDP}\;\mathrm{Standardized}\;\mathrm{Price}=\left(\frac{\mathrm{Price}\;\mathrm{Annual}}{\mathrm{GDP}\;\mathrm{per}\;\mathrm{Capita}}\right)\times 100.$$

Using the median GDP-standardized prices, a five-tier price range was created: very low, low, medium, high, and very high price. These tiers were defined by calculating the 20th, 40th, 60th, 80th, and 100th percentiles of the median prices using Excel PERCENTILE.INC formula. This formula returns the k-th percentile of values in a dataset, considering the full range of values. Products were then classified into these five price ranges accordingly.

The median value was used to define the price ranges in order to better represent the central tendency of the dataset. However, the average GDP-standardized annual price was also calculated to provide additional insight into global pricing trends for assistive products. Based on the World Bank income classification of each country listed in Table 1, the average GDP-standardized price was calculated for each income group. The average GDP-standardized annual price was also calculated for each country and each functional domain in order to adjust for the general price levels in a country when comparing across product types. The following section presents the main results of this process.

## Results

Focal points for all 12 countries submitted their price review forms, though to varying extents. Data coverage was complete or nearly complete (over 75% of products) for Brazil, Canada, China, India, Italy, Japan, Norway, and Thailand, whereas for Mozambique, Senegal, Sudan, and Zambia, coverage was below 50%. The focal point from Norway collected prices for all products. Focal points in Brazil, Canada, China, Italy, Japan, and Thailand reviewed the full list, though some products were found to be unavailable locally. The focal point in India, however, was unable to finalize the review due to time constraints.

**Figure 2:**
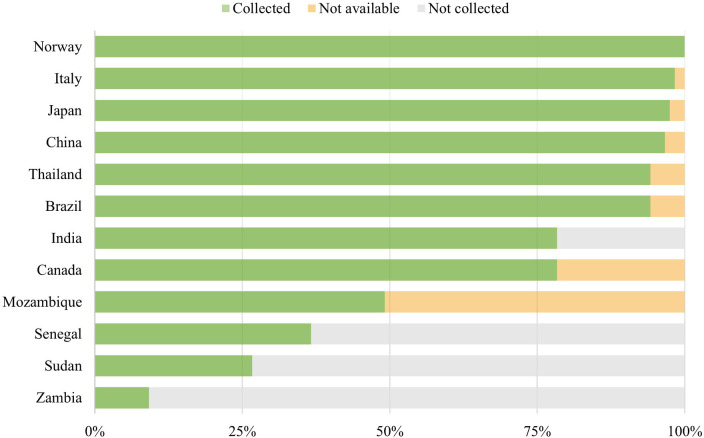
Extent of data collection for products.

In Mozambique, although all products were reviewed, more than 50% were reported as not regularly available. In Senegal, difficulties contacting suppliers limited data collection, while in Sudan and Zambia the later start of focal point activities reduced the time available to complete the review. Figure 2 illustrates the extent of data collection for each country, distinguishing between products for which prices were not collected and those reported as not available.

**Figure 3:**
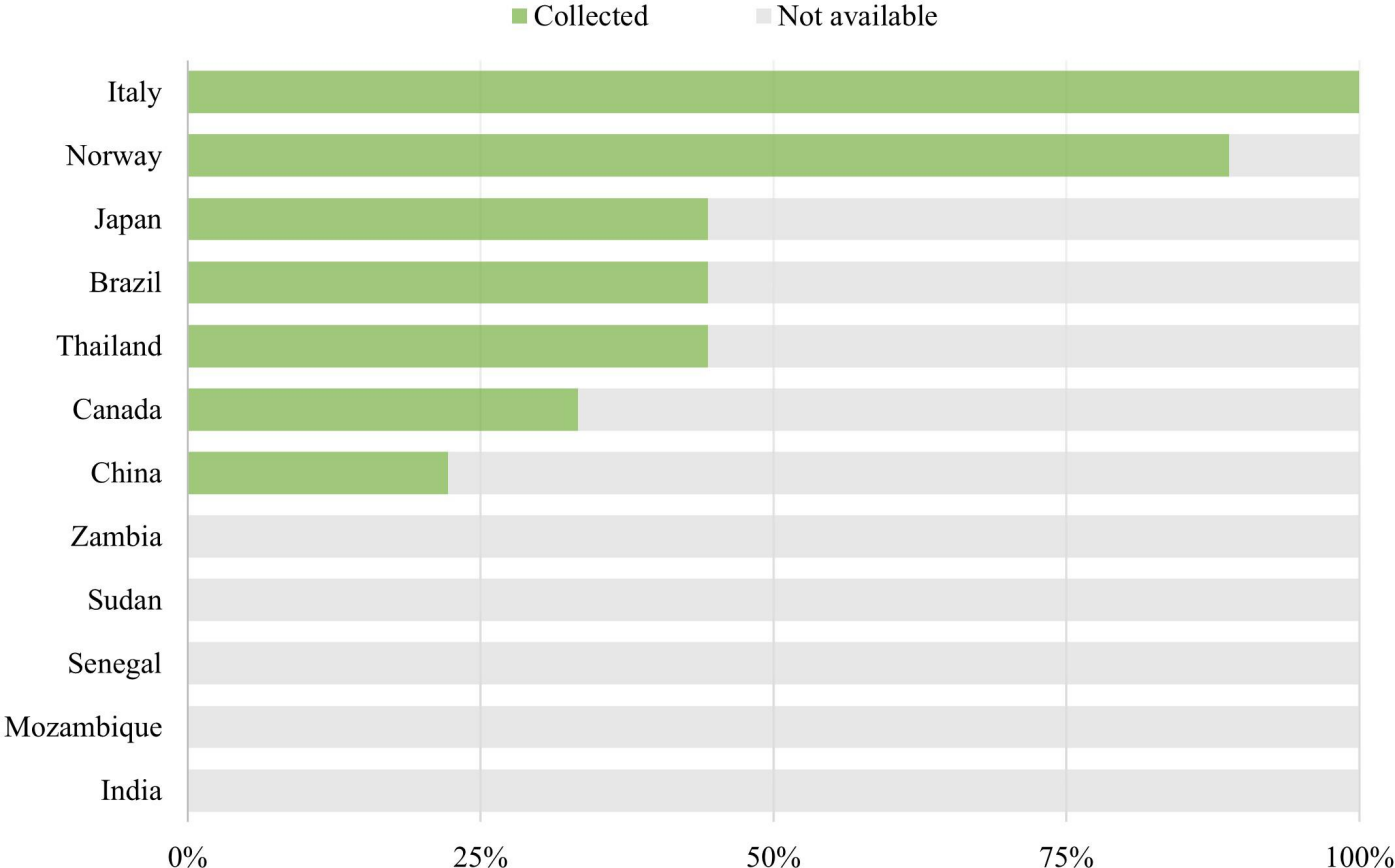
Extent of data collection for accessories.

Nine out of the 120 products required accessories. Figure 3 illustrates the extent of data collection for each country regarding accessories. Focal points working with Italy, Norway, Thailand, Brazil, Japan, Canada, and China collected prices for the applicable accessories, although to varying extents. Those working with India, Mozambique, Senegal, Sudan, and Zambia did not collect prices for accessories.

**Figure 4:**
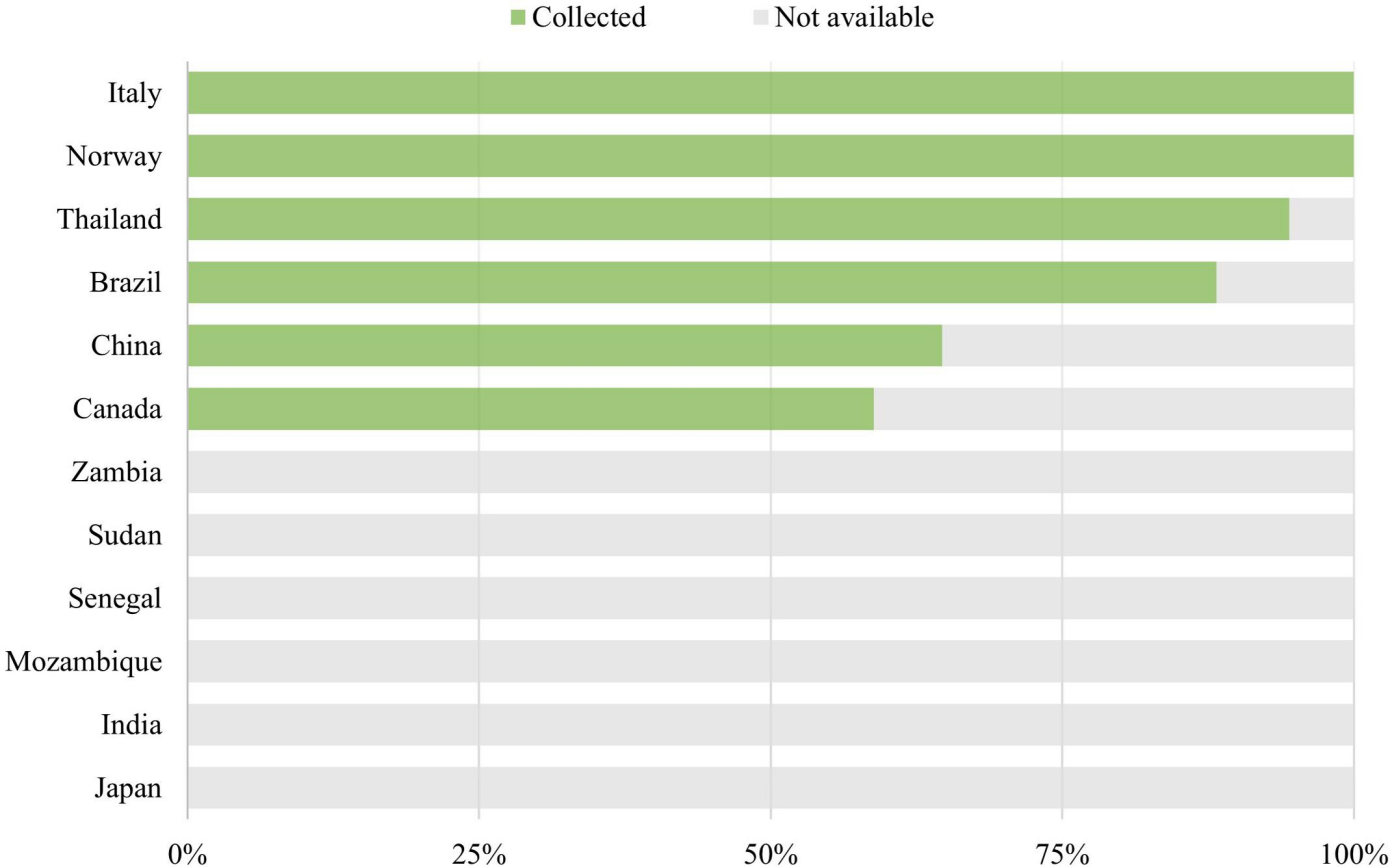
Extent of data collection for consumables.

Seventeen out of the 120 products required consumables. Figure 4 illustrates the extent of data collection for each country regarding consumables. Focal points working with Norway, Italy, Thailand, Brazil, China, and Canada collected prices for the applicable consumables, also to varying extents. Those working with Japan, India, Mozambique, Senegal, Sudan, and Zambia did not collect prices for consumables.

**Figure 5:**
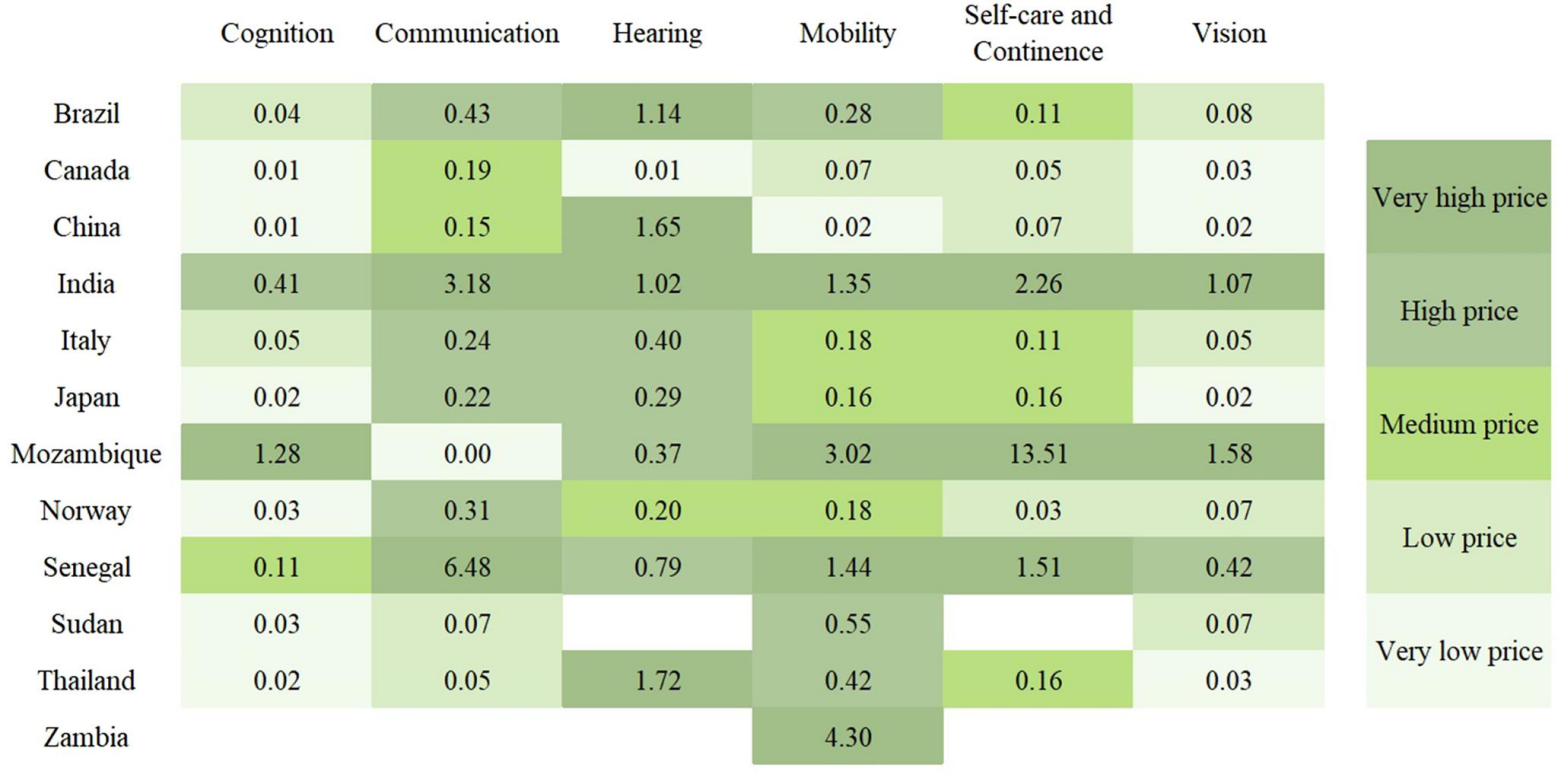
Heatmap of price ranges by country and domain (median annual price in % of GDP).

Table 2 presents the defined price ranges along with the percentage distribution of the 120 products across these ranges. Figure 5 presents a heatmap illustrating the median annual price for each country and domain, categorized according to the defined price ranges. Significant variations in pricing, both across countries and assistive product domains, were revealed.

**Table 2:** Established price ranges and product distribution.

Tiers	Percentile	Price range % GDP	Product distribution %
Very low price	0.03	≤0.03%	23%
Low price	0.1	>0.03%–≤0.1%	20%
Medium price	0.2	>0.1%–≤0.2%	15%
High price	1.0	>0.2%–≤1%	21%
Very high price	7.3	>1%	21%

**Figure 6:**
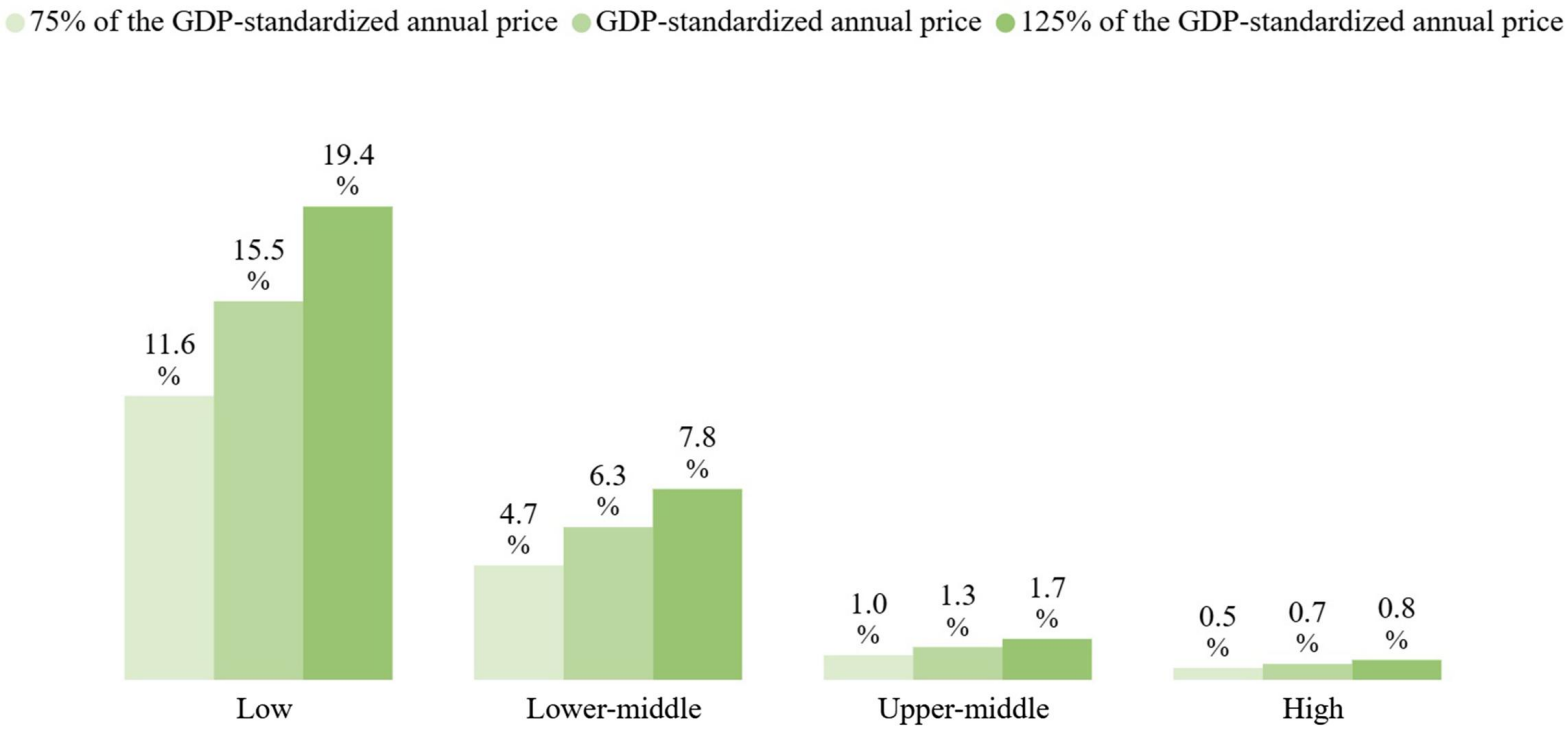
Average GDP-standardized annual price by income level.

Figure 6 illustrates the average GDP-standardized annual price by income group. An inverse relationship was found between income level and average GDP-standardized annual price—the lower the income level, the higher the average standardized price. To account for potential distortions caused by outlier GDP values, a sensitivity analysis was conducted by adjusting the average income-group GDP-standardized annual prices by +25% and −25%. As shown in Figure 6, the trend remains consistent across all income groups, indicating that the observed differences are not driven by outlier effects.

**Figure 7:**
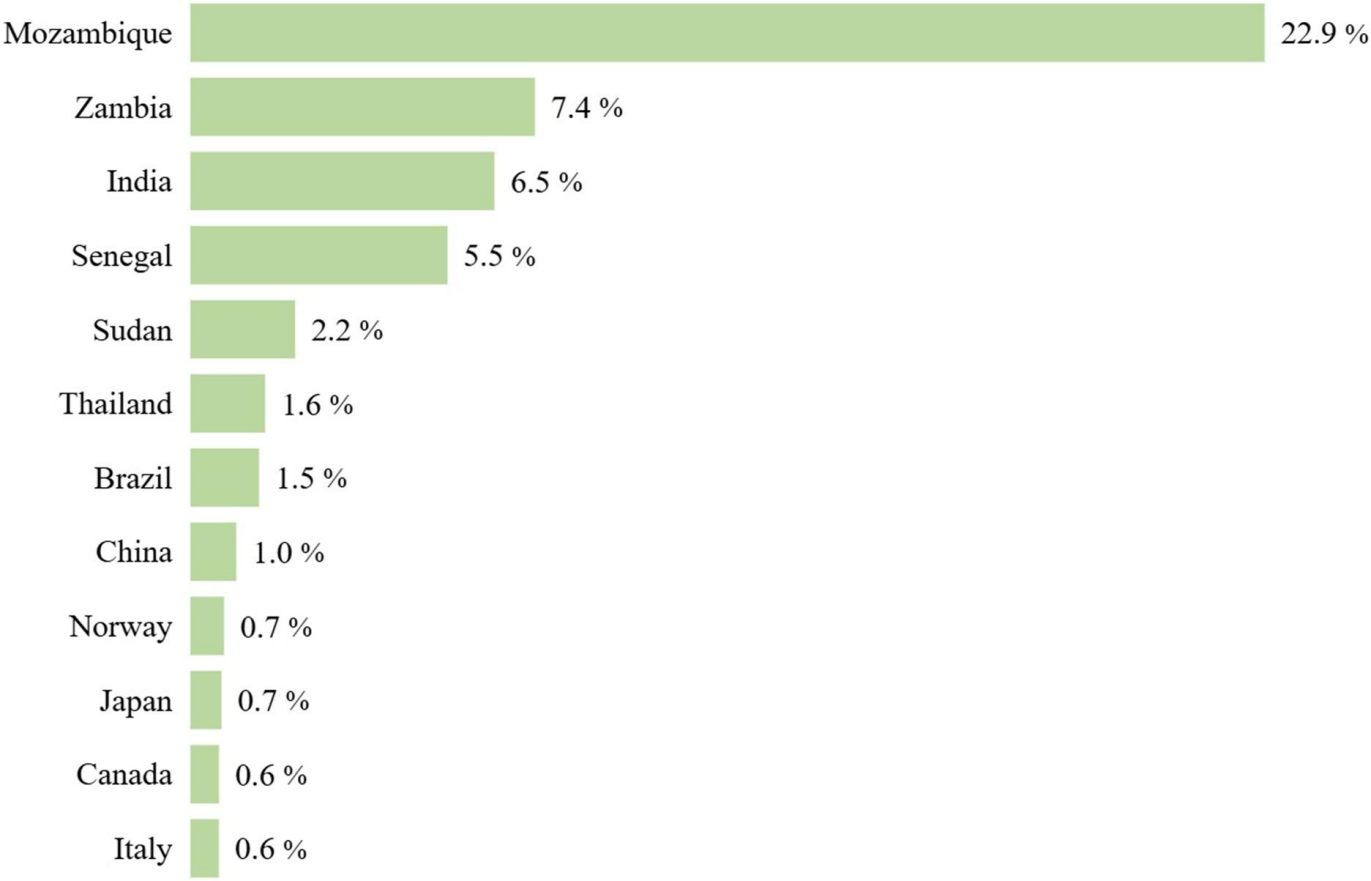
Average GDP-standardized annual price by country.

The average GDP-standardized annual price by country reinforces the pattern observed across income levels, see Figure 7. High-income countries consistently showed the lowest average GDP-standardized annual prices, while Mozambique—the country with the lowest GDP per capita in the sample—presented the highest.

**Figure 8:**
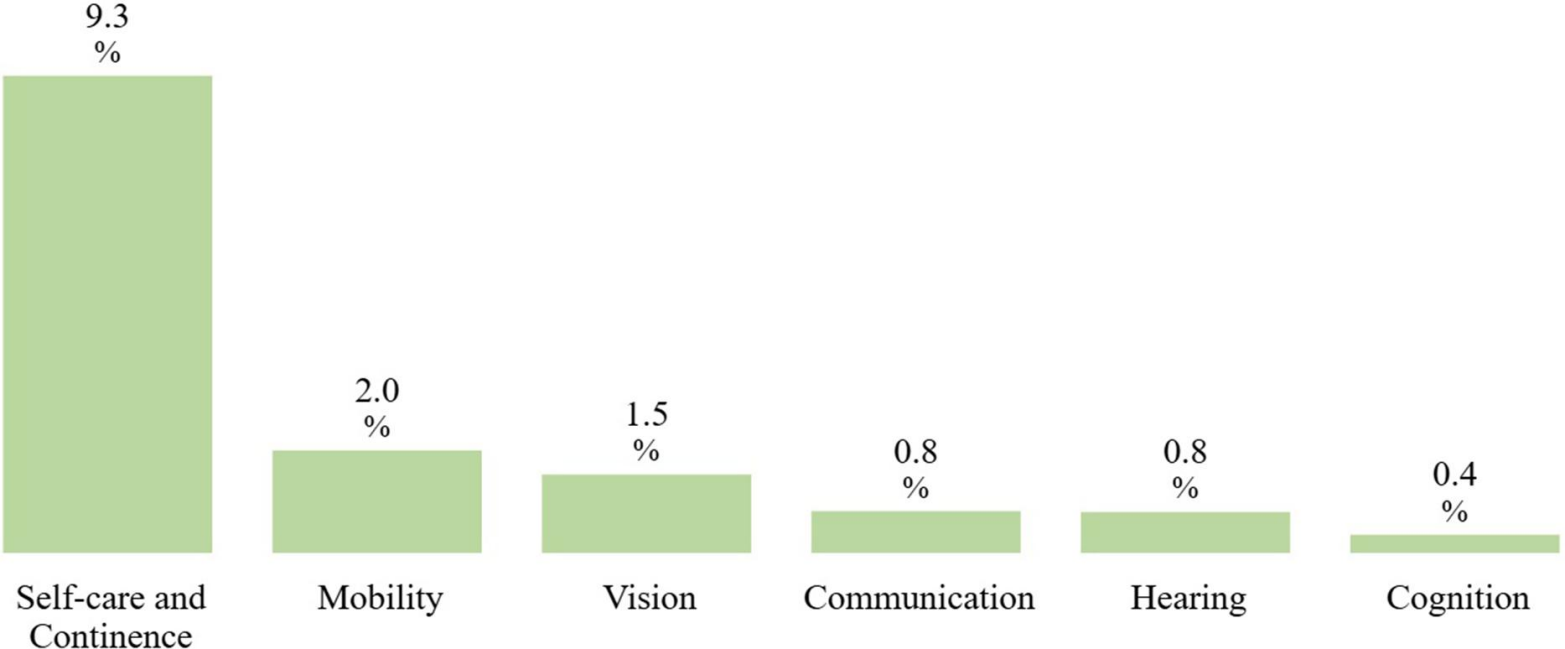
Average GDP-standardized annual price by assistive product domain.

**Figure 9:**
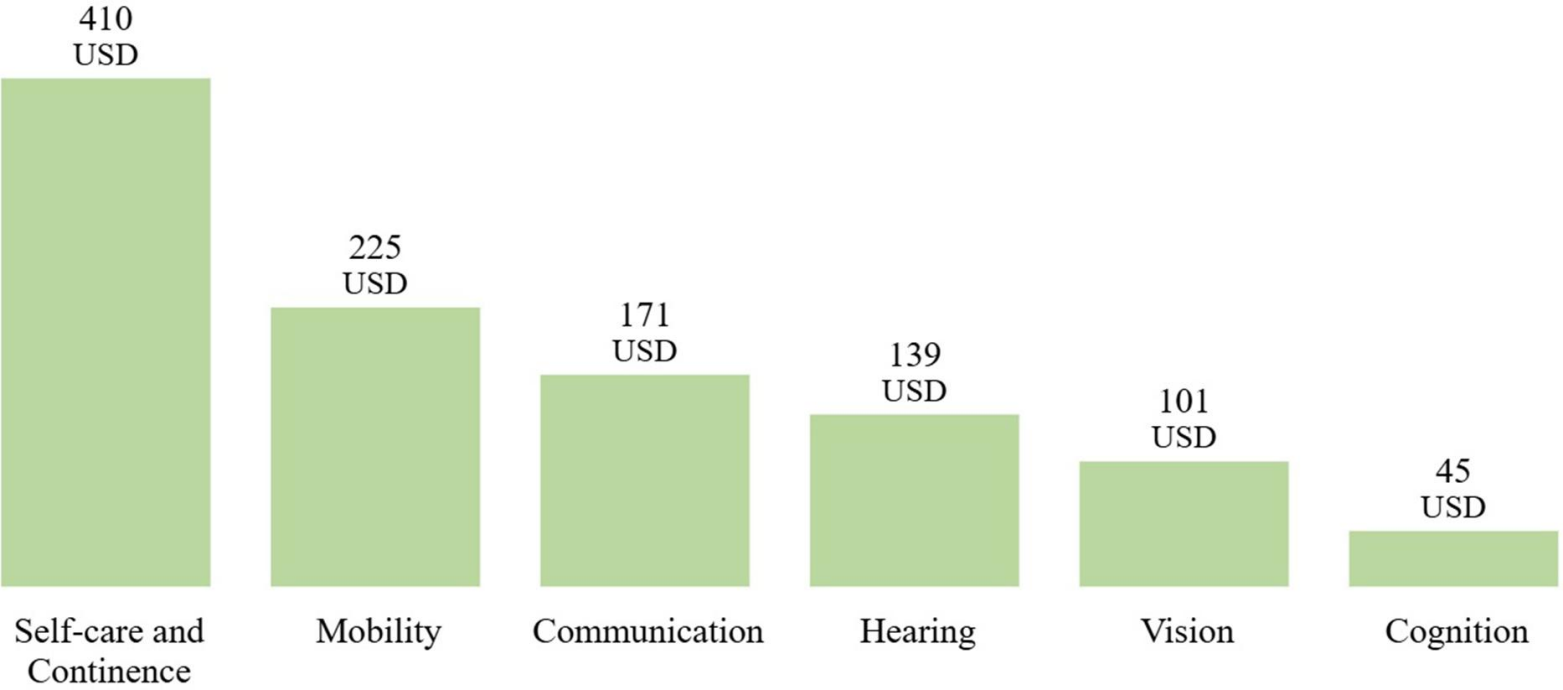
Average annual price by assistive product domain.

Among the functional domains, the self care and continence domain showed the highest average GDP-standardized annual price, at 9.3%, while the cognition domain presented the lowest, at 0.4%—indicating a significant difference in annual prices between domains. Even without GDP standardization, the self-care and continence domain remained the category with the highest average annual price (USD 410), while the cognition domain remained the lowest (USD 45). Figure 8 presents the average GDP-standardized annual price by assistive product domain, and Figure 9 presents the non-standardized average annual price.

## Discussion

The price review conducted across the 12 countries, together with the classification of each assistive product into price ranges, aimed to provide the WHO ATA team with a clearer understanding of global price levels for assistive products and inform the decision-making process for the updated APL.

The extent of data collection on products, accessories, and consumables offered valuable insights into the availability of assistive products across countries. Focal points in Brazil, Canada, China, India, Italy, Japan, Norway, and Thailand—classified as high- and upper-middle-income countries, with the exception of India—were able to collect a relatively large share of prices, whereas focal points in Mozambique, Senegal, Sudan, and Zambia, all classified as low- or lower-middle-income countries, collected fewer prices. Mozambique represented a particular case: although all products were reviewed, more than half were reported as not available in the country. Globally, the limited availability of good quality assistive products remains one of the main barriers to access to assistive technology, as highlighted by the UNICEF-WHO *Global report on assistive technology*.

The price data collected provided valuable insights into the global pricing of assistive products. When examining the average GDP-standardized annual price across countries, a clear pattern emerged: high-income countries showed the lowest prices, followed by upper-middle-income countries, while low-income countries reported some of the highest prices. This indicates an inverse relationship between income level and affordability, where the lower a country's income, the greater the financial burden of assistive products. Sudan, however, emerged as an outlier. Despite being classified as a low-income country, its price levels were closer to those of upper-middle-income countries. The reasons for this deviation remain unclear and warrant further investigation into national assistive product markets.

The analysis indicated a potential double disadvantage for countries with lower income levels: assistive products may be less available, and those that are available tend to be relatively more costly than in countries with higher income levels. This pattern is exemplified by the situations in Mozambique and Senegal, where focal points were unable to collect all prices because of limited availability of products within their countries. For other countries with missing prices, apart from Mozambique, it is uncertain whether all assistive products were in fact available, given that the shorter price-collection period for India, Sudan, and Zambia may have affected the results.

These findings align with previous research analyzing access across countries based on their Human Development Index (HDI) levels. Data for this research was drawn from a series of Rapid Assistive Technology Assessment (rATA) studies, which collected self-reported data on assistive technology access in 29 countries between April 2019 and December 2021. The rATA data demonstrated that the lower the HDI classification, the lower the prevalence of access to assistive products, with lack of available products acting as a barrier to access. Specifically, countries with a low HDI reported a median access rate of 10.7%, while those with a very high HDI reported a median access rate of 87.7% (1). It is important to point out that the rATA data reflect out-of-pocket costs borne by users, which depend on national financing mechanisms, whereas the APL price review prioritized costs incurred by governments.

In addition, the price data collected through this project provided valuable insights into pricing across assistive products domains. Among the six domains analyzed—cognition, communication, hearing, mobility, self care and continence, and vision—the self care and continence domain had the highest average GDP-standardized annual price. This finding aligns with the consumable nature of many self care products, which typically require a substantial number of items per year to meet user needs, resulting in a higher overall annual cost. A key driver of this result is Mozambique, where the average GDP-standardized annual price for self care products reached 80.9%. However, even when this outlier is excluded, the self care and continence domain still shows the highest average GDP-standardized annual price at 4.3%, followed by mobility at 1.6%. In contrast, cognition products recorded the lowest average GDP-standardized annual price. These items are generally simpler, less costly, and tend to have a longer service life.

Certain limitations of this study may affect the interpretation of the data. The sample size was limited to 12 countries, as this was the feasible number to operationalize the price collection effort, resulting in analyses based on only 2–4 countries per income group and limiting the central tendency of the findings. The incomplete data collection in 11 of the 12 countries—particularly in the low- and lower-middle-income countries—also limits the conclusions that can be drawn from the data and constrains cross-country comparisons. However, a test of the correlation between standardized price levels and the number of countries reporting a price showed no association (*r* = 0.005, *p* = .96), indicating that the standardized price levels were not related to the number of countries with missing prices.

Moreover, the diversity of sources used by focal points to collect price information—including providers, suppliers, open markets, and custom-made products—may have introduced variations in reported prices that further complicate comparability, suggesting that future studies could usefully explore possible patterns in more depth. A related issue concerns the service life of products: although focal points prioritized the search for comparable items, the reported service life often varied within the same product category across countries, which in turn affected the calculation of the annual prices. While the data nonetheless offer valuable insights into global price levels, they should be interpreted with caution.

## Conclusion

This study represents the first comprehensive pricing analysis on global affordability of assistive products undertaken as part of the APL update process, and the first published research of its kind. Given the innovative nature of this effort and the diversity of participating countries across regions and income levels, some challenges in data collection were anticipated. Despite difficulties encountered by some focal points in identifying comparable products and collecting price data, all submitted their price review forms—although to different extents. The data gathered enabled the classification of selected assistive products into price ranges and supported additional analysis on global pricing of assistive products. These insights contributed to a broader understanding of price trends and disparities worldwide. It is hoped that such pricing analyses will continue to be developed, further expanding the global knowledge on affordability of assistive products.

## Data Availability

Data on assistive product costs may be requested from WHO at assistivetechnology@who.int.
