# Supplementary material for: A review of assistive product prices in 12 countries
Source: Front Rehabil Sci. 2026 Apr 29;7:1736102. doi: 10.3389/fresc.2026.1736102 (PMC13167995; doi:10.3389/fresc.2026.1736102)
Supplement: Supplementary file 1 [file Table1.pdf]

Supplementary Table 1. Priority assistive products selected for the price collection.

ISO product division	Reference product	Domain
04 33 03 Seat cushions and underlays for tissue integrity	Wheelchair cushion	Mobility
04 33 06 Assistive products for tissue integrity when lying down	Anti-decubitus mattress	Mobility
04 48 39 Standing frames and supports for standing	Adult standing frame	Mobility
06 03 07 Thoracic orthoses	Boston brace	Mobility
06 03 12 Cervical orthoses	Cervical collar with chin support	Mobility
06 04 03 Abdominal muscle supports	Abdominal binder	Mobility
06 03 15 Cervico-thoracic orthoses	Cervico-thoracic orthosis	Mobility
06 06 03 Finger orthoses	Finger orthosis	Mobility
06 06 07 Hand-finger orthoses	Hand-finger orthosis	Mobility
06 06 12 Wrist-hand orthoses	Wrist-hand orthosis	Mobility
06 06 15 Elbow orthoses	Elbow orthosis	Mobility
06 12 12 Knee-ankle-foot orthoses	Cruropodalic orthosis	Mobility
06 12 06 Ankle-foot orthoses	Dynamic ankle orthosis	Mobility
06 18 09 Transradial prostheses	Transradial prosthesis	Mobility
06 12 13 Lower leg orthoses	Ankle foot orthosis	Mobility
06 18 15 Transhumeral prostheses	Transhumeral prosthesis	Mobility
06 18 03 Partial hand prostheses	Silicone prosthetic fingers	Mobility
06 24 09 Transtibial prostheses	Transtibial prosthesis	Mobility
06 24 15 Transfemoral prostheses	Transfemoral prosthesis	Mobility
12 06 03 Walking frames	Walker with two front wheels	Mobility
12 22 09 Single-side manual drive wheelchairs	Hemiplegic wheelchair	Mobility
12 03 03 Walking sticks	Adjustable aluminium stick	Mobility
12 03 06 Elbow crutches	Double adjustable crutch	Mobility
12 03 21 Lateral support frames	Hemiplegic walker	Mobility

12 06 06 Rollators	Rollator	Mobility
12 18 09 Hand-propelled tricycles and quadricycles	Hand-propelled tricycle	Mobility
12 22 03 Bimanual handrim-drive wheelchairs	Bimanual handrim-drive wheelchair	Mobility
12 22 18 Push wheelchairs	Powerpush transport wheelchair	Mobility
12 23 06 Electrically powered wheelchairs with electronic steering	Powered wheelchair	Mobility
12 27 07 Prams and buggies	Baby carriage	Mobility
12 31 03 Assistive products for sliding and turning	Transfer board	Mobility
12 31 18 Carrying chairs, carrying harnesses and carrying baskets	Transfer chair	Mobility
12 36 03 Mobile hoists for transferring a person in sitting position with sling seats	Mobile hoist	Mobility
12 36 18 Stationary free-standing hoists	Autonomous single-rail system	Mobility
18 03 06 Reading tables, desks and stands	Ergo table	Mobility
18 15 Assistive products for height adjustment of furniture	Furniture raiser	Mobility
18 12 24 Separate adjustable back supports and leg supports for beds	Positioning cushion	Mobility
18 18 03 Handrails and support rails	Handrail	Mobility
18 18 06 Fixed grab bars and handgrips	Fixed support bar	Mobility
18 30 15 Portable ramps	Folding wheelchair ramp	Mobility
22 29 06 Personal emergency alarm systems	Personal emergency alarm system	Mobility
24 18 03 Devices for grasping	Tool for gripping and grasping	Mobility
24 18 06 Grip adapters and attachments	Pencil grip adapter	Mobility
24 18 27 Arm supports to permit manual activities	Assistive feeding device	Mobility
24 21 03 Manual gripping tongs	Device for reaching at a distance	Mobility

24 24 03 Fixed-position systems	Monitor arm stand for desks	Mobility
24 27 Assistive products for fixation	Assistive device for fixation	Mobility
24 36 Assistive products for carrying and transporting	Drip stand	Mobility
30 09 27 Assistive products for racquet and paddle sports	Angled aid hand and strap	Mobility
30 09 33 Assistive products for swimming and water sports	Float water walker	Mobility
30 09 37 Equipment for track and field	Racing wheelchair	Mobility
30 12 Assistive products for playing and composing music	Five-in-one battery-operated drum-set	Mobility
30 15 Assistive products for producing photos, films and videos	All-in-one mobile video kit	Mobility
30 24 06 Assistive products for fishing	Adapted electric fishing reel	Mobility
30 03 03 Toys	Speed cube toy with touch twist patch	Vision
22 09 03 Voice generators	Battery-powered artificial larynx	Communication
22 09 12 Assistive products for voice training and speech training	Talk pad communication system	Communication
22 21 03 Letter and symbol sets and boards	Communication board	Communication
22 21 12 Face-to-face communication software	Communication software	Communication
22 24 24 Software for distant communication	Software for distant communication	Communication
22 06 12 In-the-ear hearing aids	In-the-ear hearing aid	Hearing
22 06 15 Behind-the-ear hearing aids	Behind-the-ear hearing aid	Hearing
22 06 25 Sound transmission systems for hearing aids	Sound transmission systems for hearing aids	Hearing
22 21 21 Assistive products for training in sign language	Pocket translator app	Hearing
04 19 04 Assistive products for administering non-liquid medicines	Pill organizer	Cognition
22 15 12 Assistive products for training and	Wooden abacus	Cognition

supporting the basics of arithmetic		
24 13 05 Wireless remote controls	Wireless remote control	Cognition
22 15 06 Calculators	Desk calculator with large display	Vision
22 28 03 Clocks and timepieces	Alarm clock for bedroom	Cognition
22 28 06 Calendars and timetables	Alzheimer calendar clock	Cognition
22 29 12 Localization and tracking systems	Localization and tracking system for people with dementia	Cognition
24 06 Assistive products to manipulate containers	Container opener for weak hands	Mobility
24 09 03 Push-buttons	Switch button for accessibility and special needs	Mobility
24 13 24 Software for operating electrical devices	Keyboard and mouse emulation software	Mobility
24 13 18 Keyboards	Large print computer keyboard	Vision
24 13 21 Computer pointing devices	Adaptive mouse	Mobility
27 06 Measuring instruments	Braille 15 ft steel tape measure	Vision
30 03 09 Games	Tactile dominoes	Vision
12 08 03 Guide canes	Aluminum mobility folding cane for vision impaired	Vision
22 03 09 Magnifier glasses, lenses and lens systems for magnification	Standing illuminated magnifier	Vision
22 13 15 Text-to-speech devices and software	eBook reader	Vision
22 13 21 DAISY players and e-book readers	Software e-book reader	Vision
22 13 27 Manual Braille writing equipment	Braille slate with clipboard	Vision
22 13 36 Portable note-taking devices for Braille	Braille display	Vision
22 15 15 Assistive products for training in basic geometric skills	Colour and shape sorter puzzle	Vision
09 06 06 Assistive products for eye protection or face protection	Eye patch	Vision
22 13 39 Text and multimedia processing	Screen reader	Vision

software		
22 39 04 Visual displays	Optical acrylic screen filter	Vision
22 45 03 Assistive products for electronic orientation	Talking orientation device	Vision
22 45 06 Assistive products for acoustic navigation	Walking cane with sound and light voice alarm	Vision
30 09 03 Assistive products for team ball sports	Goalball equipment	Vision
30 18 03 Tools, materials and equipment for textile handicraft	Threading helper	Vision
30 27 Assistive products for camping and caravanning	Nordic walking stick	Vision
30 34 Assistive products for care of animals	Long handle pooper scooper	Vision
22 21 09 Dialogue units	Multi-level communicator for speech therapy, special needs, and autism support	Communication
22 03 03 Light filters (absorption filters)	Filtering lenses	Vision
22 03 06 Spectacles and contact lenses	Ordinary spectacles	Vision
22 29 03 Signalling devices	Visual alert system	Hearing
09 06 09 Assistive products for ear protection or hearing protection	Hearing protection headphone	Hearing
09 06 21 Assistive products for heel protection, toe protection or foot protection	Heel protectors for pressure sores in bed	Self care and continence
09 07 06 Positioning pillows, positioning cushions and positioning systems	Positioning wedge pillow for side sleeping	Self care and continence
09 09 03 Assistive products for putting on or removing clothing	Sock assistance device	Self care and continence
09 09 06 Shoehorns and bootjacks	Long handled shoe horn	Self care and continence
09 12 03 Commode chairs	Bedside commode seat	Self care and continence
09 12 06 Toilets	Raised toilet seat with handles and open-front design	Self care and continence
09 12 33 Bedpans	Bedpan	Self care and continence
09 24 03 Long-term indwelling catheters	Urinary incontinence kit	Self care and continence
09 24 06 Intermittent catheters inserted through	Intermittent catheters	Self care and continence

the urethra	inserted through the urethra	
09 24 09 Penile sheaths	Wearable urinal bag with reusable silicone urine catheter bag	Self care and continence
09 30 13 Products for children with fastener system, single-use	Children diapers	Self care and continence
09 30 18 Products insert type, single-use	Large disposable pads for urinary incontinence	Self care and continence
09 30 21 Products with fastener system, single-use	Unisex adult absorbent underwear, single-use	Self care and continence
09 30 26 Pads to contain urine, with adhesive strip, single use	Pads to contain urine, with adhesive strip, single use	Self care and continence
09 30 45 Underpads for non-body-worn use to protect chair or bedding, washable	Reusable incontinence bed pads	Self care and continence
09 27 09 Non-body-worn urinals and urine bottles	Urinals for men	Self care and continence
09 31 09 Incontinence alarms	Incontinence and bedwetting pad alarm	Self care and continence
09 33 07 Shower chairs with and without wheels	Shower chair without wheels	Self care and continence
09 54 12 Assistive products for sexual habilitation and rehabilitation	Unisex, external use vibrator	Self care and continence
15 09 21 Food guards	Raised edge for plate	Self care and continence
09 12 24 Toilet arm supports and toilet back supports mounted on toilet	Toilet safety rail height adjustable	Self care and continence
